# Altered brain functional dynamics in auditory and visual networks in schizophrenia

**DOI:** 10.1192/j.eurpsy.2021.428

**Published:** 2021-08-13

**Authors:** W.F. You, L. Luo, F. Li, Q. Gong

**Affiliations:** Huaxi Mr Research Center, West China Center of Medical Sciences, Sichuan University, Chengdu City, Sichuan Province, China

**Keywords:** hallucination, dynamic functional connectivity, eigenvector centrality, schizophrénia

## Abstract

**Introduction:**

One of the most perplexing and characteristic symptoms of the schizophrenia (SZ) patients is hallucination. The occurrence of hallucinations to be associated with altered activity in the auditory and visual cortex but is not well understood from the brain functional network dynamics in SZ.

**Objectives:**

To explore the brain abnormal basis of hallucinations in SZ with the dynamic functional connectivity (dFC).

**Methods:**

Using magnetic resonance imaging for 83 SZ patients and 83 matched healthy controls and independent component analysis, 52 independent components (ICs) were identified as nodes and assigned into eight intrinsic connectivity networks (Figure 1A). Subsequently, we established dFC matrices and clustered them into four discrete states (Figure 1B) and three state transition metrics were obtained. To further explore the changes in the centrality of each component, eigenvector centrality (EC) was calculated and its time-varying was evaluated.

**Figure:**
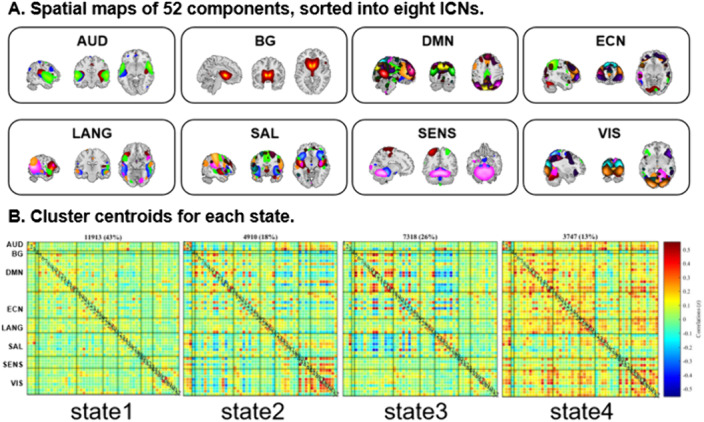

**Results:**

Compared to controls with FDR correction, we found that patients had more mean dwell times and fractional time in state 1 (P=0.0081 and P=0.0018), mainly with hypoconnectivity between auditory and visual network and other networks and hyperconnectivity between language and default-mode network (DMN). While, patients had less dwell times and fractional time in state 3 (P=0.0018 and P=0.0009), and decreased FC between visual network and executive control network (ECN) and increased FC between ECN and DMN than controls (Figure 2).

**Figure:**
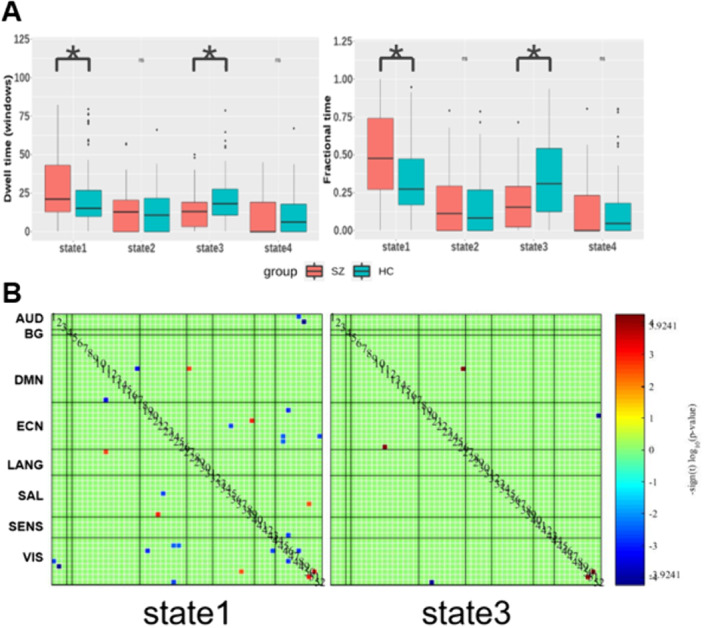

EC statistics showed that SZs displayed increased temporal dynamics in visual-related regions (Figure 3).

**Figure:**
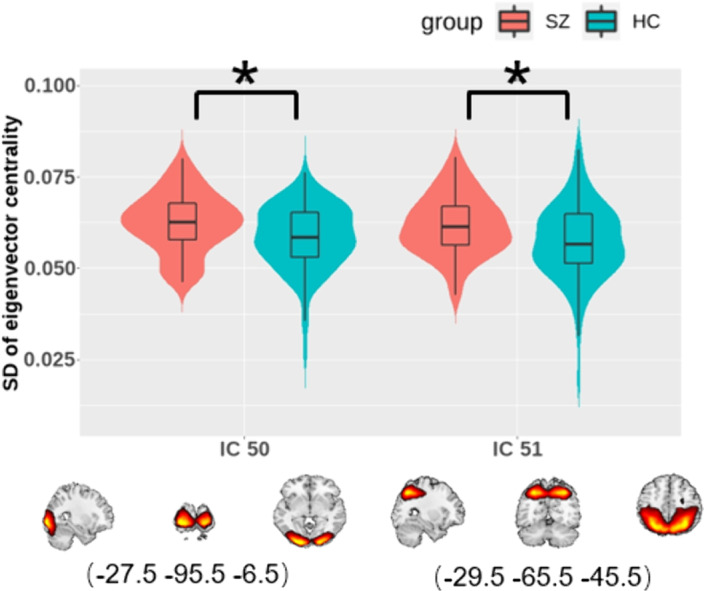

**Conclusions:**

SZ was mainly manifested as altered dFC and temporal variability of nodal centrality in auditory and visual networks.

**Disclosure:**

No significant relationships.

